# Once-weekly teriparatide improves glucocorticoid-induced osteoporosis in patients with inadequate response to bisphosphonates

**DOI:** 10.1186/s40064-016-2704-5

**Published:** 2016-07-11

**Authors:** Takahiro Seno, Aihiro Yamamoto, Yuji Kukida, Aiko Hirano, Takashi Kida, Amane Nakabayashi, Kazuki Fujioka, Hidetake Nagahara, Wataru Fujii, Ken Murakami, Ryo Oda, Hiroyoshi Fujiwara, Masataka Kohno, Yutaka Kawahito

**Affiliations:** Inflammation and Immunology, Graduate School of Medical Science, Kyoto Prefectural University of Medicine, Kajii-cho, Kawaramachi-Hirokoji, Kamigyo-ku, Kyoto, 602-8566 Japan; Department of Orthopaedics, Graduate School of Medical Science, Kyoto Prefectural University of Medicine, Kajii-cho, Kawaramachi-Hirokoji, Kamigyo-ku, Kyoto, 602-8566 Japan

**Keywords:** Teriparatide, Once-weekly, Glucocorticoid, Osteoporosis, Bisphosphonate

## Abstract

**Background:**

Patients with glucocorticoid-induced osteoporosis (GIOP) are at very high risk of fracture, and patients with severe GIOP often experience fractures during treatment with bisphosphonates. Teriparatide (TPTD) is the only currently available anabolic agent expected to be effective for GIOP. Once-weekly TPTD decreased bone resorption marker with primary osteoporosis different from daily TPTD, but it has not yet been tested with GIOP.

**Objectives:**

To evaluate the efficacy of once-weekly TPTD for patients with GIOP and inadequate response to bisphosphonates.

**Methods:**

Patients with GIOP and collagen diseases treated with prednisolone for at least 6 months with inadequate responses to bisphosphonates were administered once-weekly TPTD. Bone density of the lumbar spine and femoral neck, measured as percent young adult mean (YAM); serum concentrations of cross-linked N-terminal telopeptides of type I collagen (NTx), bone alkaline phosphatase (BAP), and calcium; and FRAX were measured at baseline and 6, 12 and 18 months after starting TPTD.

**Results:**

Of the 12 GIOP patients with collagen diseases enrolled, nine (seven females, two males; mean age 57.4 ± 11.1 years) completed treatment, including six with systemic lupus erythematosus, two with rheumatoid arthritis, and one with adult onset still disease. Only one new fracture event, a lumbar compression fracture, occurred during the study period, although seven patients experienced eight fracture events within 18 months before starting TPTD (*p* = 0.04). Lumbar spine YAM significantly improved at 18 months (*p* = 0.04), whereas femoral neck YAM did not (*p* = 0.477). Serum NTx, BAP, Ca, and FRAX were not significantly affected by TPTD treatment.

**Conclusions:**

Once-weekly TPTD reduces fracture events and increases bone density of the lumbar spine of GIOP patients with inadequate response to bisphosphonates.

## Background

Glucocorticoids are widely utilized to treat many collagen diseases. Due to their extensive use, glucocorticoid therapy is the most common cause of secondary and drug-induced osteoporosis. The pathophysiology of glucocorticoid-induced osteoporosis (GIOP) is multi-factorial, with osteoblastic dysfunction being an important aspect in the mechanism of glucocorticoid effects (Dalle Carbonare et al. 2005; (Patschan et al. 2001). Moderate doses of oral glucocorticoids inhibit synthesis of bone collagen by osteoblasts and the conversion of precursor cells into functioning osteoblasts (Patschan et al. 2001). Moreover, oral glucocorticoids were shown to have direct pro-apoptotic effects on osteoblasts in a mouse model (O’Brien et al. 2004). Glucocorticoids also modify the synthesis, release, and receptor binding of locally produced growth factors (Canalis 1996). These adverse effects of glucocorticoids on bone formation reduce the total amount of bone (Dalle Carbonare et al. 2005). Agents that promote bone formation are needed for patients with GIOP.

Teriparatide (TPTD), a human parathyroid hormone analog, is the only currently available anabolic agent that stimulates osteoblast activity (Borggrefe et al. 2010); (Sibai et al. 2011). TPTD has been shown to increase bone mass, improve bone quality, and reduce the risk of fracture in patients with severe osteoporosis (Toulis et al. 2011). Daily administration of recombinant TPTD has been shown to be effective in the treatment of nonunion fractures (Oteo-Alvaro and Moreno 2010); (Lee et al. 2012). Daily TPTD increased bone mineral density more than alendronate in GIOP (Saag et al. 2007). Although assays of markers of bone metabolism showed that daily TPTD increased markers of bone formation and bone resorption, the balance favored bone formation (Miyauchi et al. 2010).

Once-weekly chemically synthesized TPTD has been shown to have rapid and powerful anti-fracture activity (Nakamura et al. 2012); (Nakano et al. 2014), increasing the bone mineral density (BMD) of cancellous but not cortical bone (Sone et al. 1995). Once-weekly TPTD significantly reduced NTx levels from baseline by 12.2 % at 48 weeks and thereafter (Nakamura et al. 2012). This reduction of bone resorption activity is the unique feature of once-weekly TPTD although daily TPTD increase bone resorption marker (McClung et al. 2005). Even after treatment with bisphosphonates, once-weekly TPTD injections reduced the risk of vertebral fracture and increased BMD with primary osteoporosis subjects. Glucocorticoid promotes bone resorption activity in addition to the reduction of bone formation. Therefore, these findings suggested that once-weekly TPTD may be effective in patients with GIOP. This pilot study assessed the efficacy of once-weekly TPTD for GIOP patients with inadequate response to bisphosphonates.

## Methods

### Study design

The study cohort consisted of patients at our institution with GIOP and collagen diseases, who showed inadequate responses to bisphosphonates. Patients received subcutaneous injections of 56.5 μg of TPTD once-weekly for 18 months. During the study, all the patient concomitantly received vitamin D. Markers of osteoporosis were assessed at baseline and after 6, 12 and 18 months of treatment. The study protocol was approved by the responsible institutional review boards at our hospital (RBMR-C-1154-3), was conducted in accordance with the ethical standards of the Declaration of Helsinki, and was consistent with good clinical practice.

### Participants

Osteoporosis was diagnosed according to the guidelines on the management and treatment of GIOP of the Japanese Society for Bone and Mineral Research (Nawata et al. 2005), i.e., with BMD at the lumbar spine or femoral neck <80 % of the young adult mean (YAM) in the Japanese population. Moreover, all patients were treated with corticosteroids for at least 6 months, but experienced new fractures or decreased BMD despite treatment with bisphosphonates, after which patients were started on once-weekly TPTD instead of bisphosphonates. All patients provided written informed consent.

### Efficacy measures

Bone mineral density of the lumbar spine and hip was measured in nine and six subjects, respectively, using dual-energy x-ray absorptiometry (Discovery Wi; Hologic, Bedford, MA) at baseline and at 6, 12 and 18 months. BMD and YAM were determined, and changes from baseline were calculated.

Serum samples were obtained under nonfasting conditions before injection of TPTD. All measurements were performed centrally in a single batch at our hospital and a validated institution (SRL, Tokyo, Japan). Serum concentrations of the bone formation marker bone alkaline phosphatase (BAP); the bone resorption marker cross-linked N-telopeptide of type I collagen (NTx), and calcium were measured at baseline and after 6, 12 and 18 months. Serum BAP was measured using a chemiluminescent enzyme immunoassay and serum NTx by enzyme-linked immunoassay.

### Fractures

Morphometric fractures were assessed in frontal and lateral spine, and hip radiographs were obtained at baseline and after 6, 12 and 18 months. Clinical fractures were defined as those confirmed on radiographs, accompanied by clinically evident symptoms such as pain in the vertebral or non-vertebral region. Both morphometric and clinical fractures were counted as fracture events.

The fracture risk assessment tool (FRAX) is a scientifically validated risk assessment tool endorsed by the World Health Organization that assesses the 10-year probability of fracture. Ten-year probabilities of hip and major osteoporotic fractures were calculated using the official calculation web tool of the World Health Organization.

### Adverse events

Patients underwent regular physical examinations, hematological monitoring, measurements of blood chemistry, and urinary examinations. All adverse events that led to withdrawal from the study were recorded.

### Statistical analysis

Percent changes from baseline in BMD, YAM, FRAX, serum calcium, and bone turnover markers at each time point were compared with baseline measurements using ANOVA and Wilcoxon matched-pairs single rank tests. All statistical analysis were performed using GraphPad Prism statistical software (Version 5, GraphPad Software, San Diego, CA, USA), and all had a two-sided significance level of 0.05.

## Results

### Subjects

Twelve GIOP patients with collagen disease were enrolled into this study, including six with systemic lupus erythematosus (SLE), three with rheumatoid arthritis (RA), one with mixed connective tissue disease, one with systemic sclerosis, and one with adult onset still disease (AOSD). One patient stopped TPTD after the first dose due to nausea, and two discontinued within 6 months because of onset of another disease unassociated with TPTD, including one patient with malignant lymphoma and the other with purulent arthritis of the hip and abscess in the iliopsoas muscle. The remaining nine patients completed this study; their baseline demographic and clinical characteristics, including concentrations of bone metabolic markers, are shown in Table 1. The nine patients, of mean age 57.4 years, included six with SLE (five females and one male), two with RA (one female and one male), and one female with AOSD. At baseline, their mean BMD was 0.74 at the lumbar spine and 0.62 at the femoral neck, and their mean YAM at these two sites was 73.1 and 72.0 %, respectively. Prior to starting TPTD treatment, seven of the nine subjects experienced vertebral or non-vertebral fractures, including seven who experienced fractures within 18 months before this study, regardless of their BMD. In remaining two patients, BMD decreased in spite of bisphosphonate treatment. Mean BAP at baseline was 10.8 µg/L and mean serum NTx was 13.8 nmol BCE/L. All patients received the administration of vitamin D. Disease activities including DAS28-CRP and SLEDAI were not changed during study period.

**Table 1:** Baseline characteristics of patients who received once-weekly teriparatide injections for 18 months

Sex (n)	Female: 7, male: 2
Age (year)	57.4 ± 11.1
Disease (n)	SLE 6, RA 2, AOSD 1
PSL dose at baseline (mg/day)	10 ± 6.6
Fracture events within 18 months before TPTD treatment (n)	8 events of 7 patients
Lumbar spine YAM (%)	73.1 ± 11.9
Total hip YAM (%)	72 ± 10.0
Lumbar spine BMD	0.74 ± 0.11
Total hip BMD	0.62 ± 0.11
Ca (mg/dl)	9.1 ± 0.5
BAP (µg/L) Reference value; male 3.7–20.9, Premenopausal female 2.9–14.5, Postmenopausal female 3.8–22.6	10.8 ± 4.6
Serum NTx (nmol BCE/L) Reference value; Male 9.5–17.7, Premenopausal female 7.5–16.5, Postmenopausal female 10.7–24.0	13.8 ± 4.4
FRAX: major fracture (%)	20.5 ± 16.7
FRAX: hip fracture (%)	8.8 ± 11.4

Data are shown as mean ± SD

*SLE* systemic lupus erythematosus, *RA* rheumatoid arthritis, *AOSD* adult onset still disease, *PSL* prednisolone, *YAM* young adult mean, *BMD* bone mineral density, *Ca* calcium, *BAP* bone alkaline phosphatase, *NTx* type 1 collagen cross-linked N-telopeptide

### Bone mineral density

**Fig. 1:**
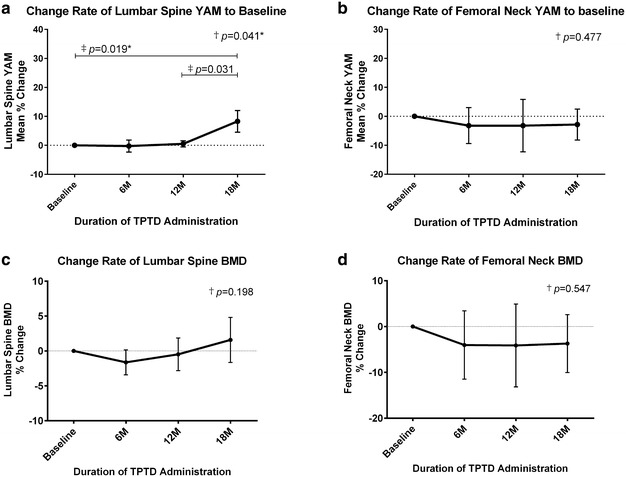
Mean percent changes in bone density from baseline during TPTD treatment. Changes over time in **a** Lumbar spine YAM; **b** Femoral neck YAM; **c** Lumbar spine BMD; **d** Femoral neck BMD. Results were compared using ^†^ANOVA and ^‡^Wilcoxon matched-pairs single rank test; *p < 0.05; *bars* indicate 95 % CI

At 18 months, YAM increased 8.27 % at the lumbar spine (*p* = 0.041, ANOVA; *p* = 0.019, Wilcoxon matched-pairs single rank test), but decreased −2.85 % at the femoral neck (*p* = 0.477, ANOVA) (Fig. 1a, b). BMD at these sites, however, did not change significantly during TPTD treatment (Fig. 1c, d), but tended to increase at the lumbar spine.

### Bone turnover markers

**Fig. 2:**
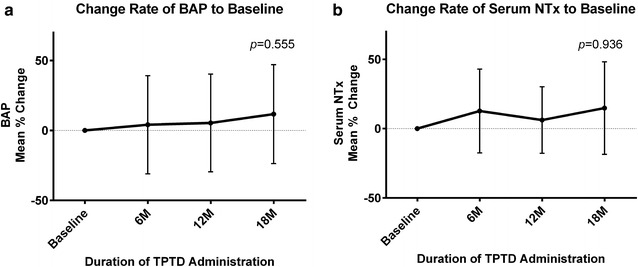
Mean percent changes in bone turnover markers from baseline during TPTD treatment. Percent changes over time in **a** mean BAP; and **b** mean serum NTx. Differences were compared using ANOVA; *bars* indicate 95 % CI. BAP was representative of bone formation marker, and NTx was for bone resorption marker

**Fig. 3:**
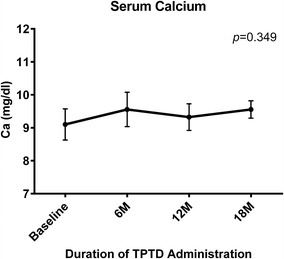
Mean serum calcium concentration during TPTD treatment. Mean serum calcium concentrations over time. Differences were compared using ANOVA; bars indicate 95 % CI

Serum BAP (Fig. 2a) and NTx (Fig. 2b) tended to increase during TPTD treatment, but not significantly. Serum calcium concentration was unchanged (Fig. 3).

### Fractures

**Fig. 4:**
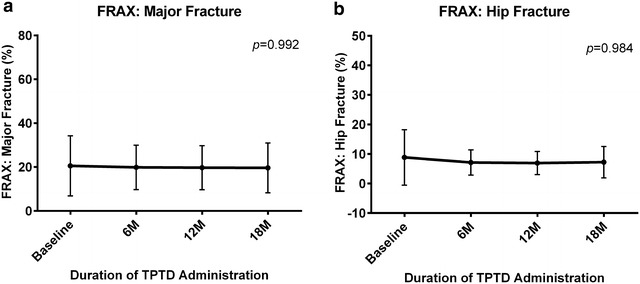
Mean FRAX score during TPTD treatment. Changes over time in FRAX scores. **a** Major fractures; and **b** Hip fractures. Differences were compared using ANOVA; *bars* indicate 95 % CI

FRAX for hip and major fractures remained constant during TPTD administration (Fig. 4a, b). The number of fracture events and patients with new fractures 18 months before or after TPTD treatment in Table 2. During the 18 months before TPTD treatment, seven patients experienced eight fracture events. During the 18 months of TPTD treatment, only one patient, a female with SLE, experienced a new radiographic vertebral fracture or investigator-assessed non-vertebral fracture (*p* = 0.04). This patient had three fracture events within 18 months before the start of the study, and showed a significant reduction from baseline in BAP concentration.

**Table 2:** Fracture events of patients who completed the 18-months study duration

Study period of TPTD administration	−18 ~ −13 months	−12 ~ −7 months	−6 ~ 0 months	0 ~ 6 months	7 ~ 12 months	13 ~ 18 months
Patients	0	1	6	0	0	1
Fracture events	0	1	7	0	0	1

This table shows the number of total fracture events and patients with new fractures before or after TPTD administration

### Safety

Of the 12 patients enrolled, nine patients completed this study. One patient discontinued TPTD due to nausea at first administration. Through 18 months, none of the patients who completed this study experienced any significant adverse events.

## Discussion

This study indicated that once-weekly TPTD was effective for subjects with GIOP who were non-responsive to bisphosphonate therapy. TPTD reduced fracture events and increased BMD at the lumbar spine. Moreover, it was indicated the new sights of bone metabolism marker change in GIOP treated with once-weekly TPTD. The mean serum concentration of NTx, a bone resorption marker, increased during treatment, although former study showed NTx levels decreased under onece-weekly TPTD treatment of primary osteoporosis (Nakamura et al. 2012).

Glucocorticoid induces osteoporosis mainly by suppressing osteoblast-mediated osteogenesis. Glucocorticoids can suppress osteoblastic differentiation and activity, both of which many be overcome by PTH and bisphosphonates (Hayashi et al. 2009). An in vitro study showed that low-dose PTH reversed glucocorticoid-inhibited alkaline phosphatase activity (Iu et al. 2005). Intermittent PTH was found to prevent glucocorticoid-induced osteoblast and osteocyte apoptosis and preserve the rate of bone formation, BMD, and strength (Weinstein et al. 2010). These findings indicate that TPTD is suitable for treatment of GIOP.

TPTD is a unique anabolic agent that accelerates bone formation. Daily TPTD has been shown to reduce bone fractures, especially in the vertebrae, increasing BMD at the lumbar spine (Reeve et al. 1976), and reducing nonunion fractures (Aspenberg and Johansson 2010). Spine and hip BMD were increased more by daily TPTD than by alendronate in patients with GIOP (Saag et al. 2009). Additionally, daily TPTD for 18 months was superior to risedronate in improving lumbar spine BMD, as measured by quantitative computed tomography, in males with GIOP (Glüer et al. 2013).

Recently, once-weekly TPTD has been available for severe osteoporosis in addition to daily TPTD. One of the most important difference between once-weekly and daily TPTD is change in bone resorption marker. Once-weekly TPTD reduces bone resorption markers in addition to increasing bone formation markers, as compared to daily TPTD that increase bone resorption markers (Miyauchi et al. 2010). Glucocorticoid also promote bone resorption. Therefore, once-weekly TPTD is thought to be more suitable for GIOP treatment than daily TPTD.

The efficacy of once-weekly TPTD had not been tested for GIOP. A double-blinded randomized clinical trial for primary osteoporosis patients confirmed that once-weekly TPTD increased lumbar BMD dose-dependently after 24 and 48 weeks, although the radiogrammetric density of cortical bone did not differ significantly (Fujita et al. 1999). This study found that once-weekly TPTD for 18 months significantly increased YAM of the lumbar spine, as well as reducing fracture events. Prior fracture is an important risk factor for future fractures (Klotzbuecher et al. 2000). In our study, TPTD prevented the fracture events in the six patients who had one fracture event during 18 months before TPTD. A female SLE patient, who had two fractures within 18 months before starting TPTD, suffered only one fracture during TPTD treatment. Furthermore she was treated with denosumab after once-weekly TPTD, she did not fracture under denosumab. Swithing from TPTD to denosumab improved bone mineral density effectively in postmenopousal osteoporosis (Leder et al. 2015). Preseding once-weekly TPTD might be beneficial for prevention of fracture. Additionally, YAM was improved at 18 months in our study, but it was not increased at 12 months. We assumed the reason that patients enrolled in this study were very severe with osteoporosis. This fact showed that we needed TPTD treatment for 18 months in order to improve GIOP. Taken together, 18 months of once-weekly TPTD reduced fractures in GIOP patients.

Our study also indicated the new findings of bone turnover makers. Assessments of bone turnover makers during once-weekly TPTD administration found that the bone formation markers BAP and osteocalcin were increased, while the bone resorption marker urinary deoxypiridinoline was decreased (Sugimoto et al. 2014), as was urinary NTx (Nakamura et al. 2012). Thus, weekly TPTD not only enhances bone formation but suppresses bone resorption. Our study found, however, that the increase in BAP was smaller than previously reported and that serum NTx increased. Although daily TPTD tended to increase serum calcium level, weekly TPTD maintained calcium concentration (Sone et al. 1995). Our study found that serum calcium did not change during 18 months of treatment with once-weekly TPTD. This study, however, included only patients with severe GIOP who were unresponsive to bisphosphonates, which may have reduced the impact of TPTD on bone turnover markers.

Moreover, lower frequency of administration decreases the burden on patients and enhance the convenience. Thus, once-weekly administration has the advantage even if its beneficial effect is equivalent to daily TPTD.

This study had several limitations, including the very small size of the study population and the evaluation of only two bone turnover markers, BAP and NTx. Other markers of bone turnover, including osteocalcin, P1NP, and deoxypiridinoline, were not measured. Third, patients did not receive supplementary calcium and magnesium as basic treatment for osteoporosis.

In conclusion, this study is the first to our knowledge to show that once-weekly TPTD reduced fracture events and increased the YAM of the lumbar spine in GIOP patients with inadequate response to bisphosphonates. Once-weekly TPTD was well tolerated and compliance was good. Although these findings suggest that TPTD may be safe and effective for GIOP patients with collagen diseases, further studies in larger patient populations are required to determine the efficacy of once-weekly TPTD for GIOP.
